# Computational Discovery of Potent Nucleoprotein Inhibitors for Influenza A Virus: Validation Through QM/MM Analysis and Experimental Binding Assays

**DOI:** 10.3390/molecules30193960

**Published:** 2025-10-02

**Authors:** Zixiao Liu, Jialin Guo, Chao Zhang, Yongzhao Ding, Shiyang Sun, Binrong Yao, Cheng Xing, Xiaoping Liu, Chun Hu, Junhai Xiao

**Affiliations:** 1 Key Laboratory of Structure-Based Drug Design & Discovery, Ministry of Education, Shenyang Pharmaceutical University, Shenyang 110016, China; lzx2023528@163.com (Z.L.); 13308109105@163.com (Y.D.); chunhu@syphu.edu.cn (C.H.); 2 State Key Laboratory of National Security Specially Needed Medicines, Beijing 100039, China; shandongguojialin@163.com (J.G.); zhangchaoylh@126.com (C.Z.); noah97sun@163.com (S.S.); binrongyao@163.com (B.Y.); xc15754368506@163.com (C.X.)

**Keywords:** nucleoprotein, influenza A virus, MM-GBSA, surface plasmon resonance, molecular dynamics

## Abstract

This study employed an integrated computational approach to discover novel nucleoprotein (NP) inhibitors for influenza A virus (IAV). Beginning with virtual screening of over 10 million compounds using Schrödinger’s Glide module (HTVS, SP, XP docking), the workflow identified promising candidates with favorable binding energies. Subsequent molecular mechanics/generalized born surface area (MM-GBSA) calculations and 100 ns molecular dynamics (MD) simulations prioritized 16 compounds for experimental validation. Surface plasmon resonance (SPR) assays revealed that compounds **8**, **13**, and **14** demonstrated superior target engagement, showing equilibrium dissociation constants (*K*_D_) of 7.85 × 10^−5^ M, 3.82 × 10^−5^ M, and 6.97 × 10^−5^ M, respectively. Molecular dynamics, alanine scanning mutagenesis, and quantum mechanics/molecular mechanics (QM/MM) analysis were conducted to analyze the binding modes, providing a reference for the design of subsequent compounds. These findings validate the efficacy of structure-based virtual screening in identifying high-affinity NP inhibitors and provide insights for the development of broad-spectrum anti-influenza therapeutics.

## 1. Introduction

Influenza poses a major threat to global health, leading to significant illness and death. Pandemics of influenza A virus (IAV), driven by novel antigenic subtypes, result in worldwide outbreaks [1]. During a pandemic year, infection rates can reach up to 50% of the population, and these events are frequently marked by a substantial surge in mortality [1]. The 20th century witnessed three major pandemics: the 1918 H1N1 (Spanish flu), the 1957 H2N2 (Asian flu), and the 1968 H3N2 (Hong Kong flu) [2]. Furthermore, the COVID-19 pandemic had already resulted in over 701,000 global fatalities and more than 18.5 million infections by early August 2020 [3]. Despite considerable scientific and technological progress and dedicated efforts, viruses persist and continue to represent a grave danger to humanity.

Influenza viruses are classified within the orthomyxoviridae family. Among these, IAV stands as the predominant cause of seasonal influenza, affecting both humans and animals [4]. The viral envelope displays three key proteins: hemagglutinin (HA), neuraminidase (NA), and the M2 proton channel [5]. IAVs are further classified based on their antigenically distinct HA and NA subtypes; 18 HA and 11 NA variants have been documented across various host species [6]. For preventing and treating influenza, the FDA currently approves two main classes of antivirals: M2 channel blockers and neuraminidase inhibitors [7]. Specifically, the IAV M2 protein (A/M2) is targeted by the licensed drugs amantadine and rimantadine [8]. However, widespread resistance to these adamantanes has developed in circulating IAV strains, severely restricting their clinical use [9]. Additional limitations include potential neurotoxicity and ineffectiveness against influenza B virus (IBV) [10,11]. The concept of targeting viral proteins for drug development is validated by zanamivir (Relenza) and oseltamivir (Tamiflu), the first approved NA inhibitors effective against both IAV and IBV [12]. Unfortunately, extensive use of these NA inhibitors has also led to the emergence of resistant viruses [13]. Consequently, developing a new generation of antiviral agents is an urgent priority.

**Figure 1:**
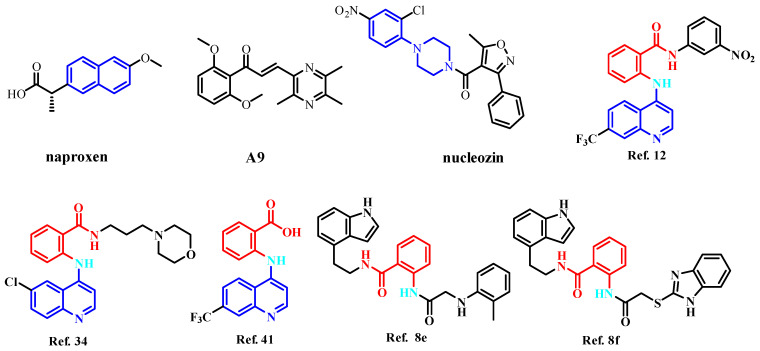
Structures of compounds that inhibit the NP domains of influenza virus [24,25].

**Figure 2:**
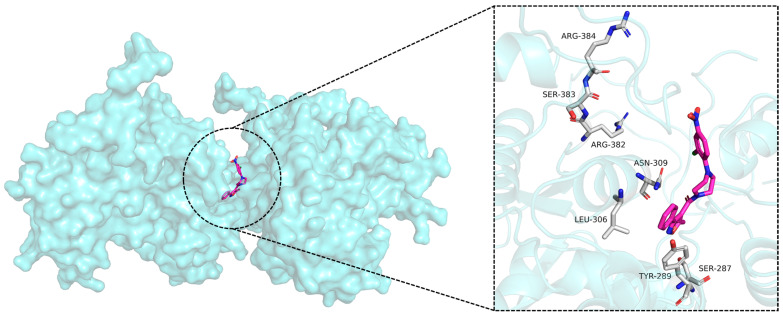
The docking poses of NP (PDB ID: 5B7B) and nucleozin.

Nucleoprotein (NP) inhibitors represent a highly promising antiviral class due to their strong antiviral effects and broad-spectrum activity [14,15]. As a viral protein, NP lacks a human cellular equivalent, enabling NP inhibitors to achieve high selectivity. Furthermore, NP exhibits significant conservation across influenza A strains from diverse species, suggesting a reduced likelihood of resistance development against NP-targeting drugs [5]. Within infected cells, NP is abundantly expressed and serves as an essential component of the viral replication cycle [16,17]. The monomeric NP is a 56 kDa protein containing a patch of basic residues that binds single-stranded RNA, with each NP monomer interacting with approximately 24 RNA nucleotides [18,19]. The combination of NP’s multifunctional roles and its high sequence conservation establishes it as an ideal target for antiviral drug development [20]. Three primary approaches exist to disrupt NP function. The first strategy focuses on inhibiting NP’s RNA-binding activity [21]. For example, naproxen (Figure 1) and its structural analogs are proposed to target NP, preventing the NP-RNA association essential for its function [21]. The second strategy aims to block the nuclear export of NP [22]. This is exemplified by the ligustrazine derivative A9 (Figure 1), which exhibits antiviral activity against influenza A and B viruses through NP binding, thereby inhibiting vRNP nuclear export [23]. The third strategy involves disrupting monomer–oligomer equilibrium by interfering with oligomerization by either promoting or inhibiting the process. The salt bridge formed between residues E339 and R416 of adjacent monomers, which is necessary for NP trimerization, was the target of NP inhibitors discovered through computational screening, such as compounds Ref. 12 [24], Ref. 34 [24], Ref. 41 [24], Ref. 8e [25], and Ref. 8f [25]. Nucleozin (Figure 1) and its derivatives have attracted considerable attention as promising anti-influenza agents [26]. Nucleozin induces the formation of large NP aggregates, prevents NP accumulation in the nucleus, and consequently halts viral replication. This demonstrates effectiveness during both the early and late phases of the viral infection cycle [27]. Figure 2 depicts the binding diagram of nucleozin to NP. Key interactions were observed between nucleozin and residues SER287, TYR289, LEU306, ASN309, ARG382, SER383, and ARG384.

**Figure 3:**
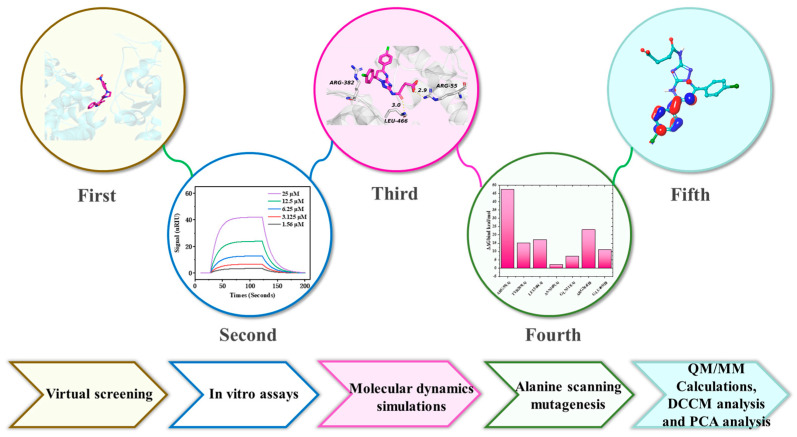
Detailed workflow of this study.

Virtual drug screening, or virtual screening for short, refers to the screening of candidate drug ligands for target receptor proteins from a large-scale compound ligand database by a computational model to predict possible potential drugs [28]. Virtual screening is of cardinal importance for in silico drug discovery processes to speed up drug development, as it helps in the selection of the best candidate drugs [29]. In this study, we aim to find potent and specific inhibitors against NP through structure-based drug design (Figure 3). Subsequently, high-throughput virtual screening (HTVS), standard precision (SP), and extra precision (XP) protocols in Schrödinger software (version 2023-1) were employed to screen potential NP inhibitors, yielding 46 candidate compounds. These compounds underwent molecular mechanics/generalized born surface area (MM-GBSA) simulations to estimate their binding free energies. Ultimately, surface plasmon resonance (SPR) assays were utilized to measure the interaction affinities of the 16 prioritized lead compounds with the NP target. Generally, we obtained three potent NP inhibitors, which provided a reference for the design of subsequent compounds.

## 2. Results and Discussion

### 2.1. Virtual Screening

In this investigation, nucleozin served as the benchmark compound due to its well-documented efficacy as a prominent NP inhibitor. This study initiated a large-scale virtual screening of over 10 million compounds, predominantly from the ChemDiv and Chembridge libraries, to discover potential NP-targeting agents. Utilizing HTVS methodology, 1356 compounds exhibiting favorable binding affinities (docking scores below −5 kcal/mol, which was similar to the docking score of nucleozin (−5.003 kcal/mol)) were initially selected. These hits subsequently underwent rigorous analysis through SP docking protocols, culminating in the identification of 148 promising candidates for further investigation that was better than nucleozin’s docking score (−6.750 kcal/mol). In the final phase, XP molecular docking simulations were conducted, yielding 46 compounds with XP GScores below the −5 kcal/mol threshold. Notably, nucleozin demonstrated robust binding performance in XP docking, registering a GScore of −4.835 kcal/mol, which quantitatively validates its superior NP-targeting capabilities compared to newly identified candidates. The physicochemical characteristics of the compounds and their interactions with the amino acids in the protein pocket, such as hydrogen bonding, hydrophobic interactions, and other kinds of interactions, are taken into account in addition to docking scores when choosing compounds. It is crucial to emphasize that experimental validation is necessary for the final screening results.

**Figure 4:**
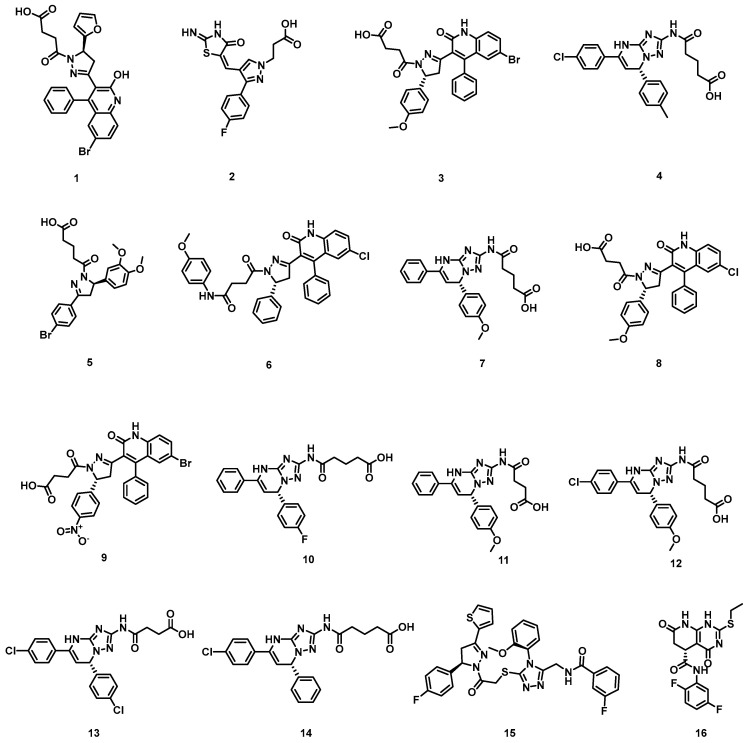
Chemical structures of identified top 16 lead molecules.

We applied the Prime MM-GBSA computational method, considered the gold standard for assessing ligand–protein binding, to determine the binding free energy profiles of 46 compounds. Nucleozin had a binding free energy of −41.83 kcal/mol. Thus, we selected compounds that exhibited similar binding free energy (−35.00 kcal/mol), with nucleozin as the candidate compound. However, only 16 compounds were identified as lead molecules (Table S1), as depicted in Figure 4. Structural characterization demonstrated that these prioritized compounds achieved optimal spatial complementarity within the protein’s catalytic pocket. All candidates exhibited favorable binding energies in our quantitative evaluation of molecular interactions (Table 1). The van der Waals (VdW) components, exceeding computational thresholds, are particularly critical for ensuring stable target binding and consequent pharmacological inhibition. Notably, molecular dynamics simulations highlighted robust intermolecular contacts with key catalytic residues TYR289(A), LEU306(A), ASN309(A), and ARG384(B), suggesting competitive binding mechanisms comparable to established NP inhibitors.

Comprehensive molecular docking metrics encompassing scoring functions, critical intermolecular contacts, structural compatibility parameters, and thermodynamic binding profiles for these prioritized compounds are detailed in Table 1. Following computational validation, the 16 lead candidates which were purchased from TargetMol Chemicals Inc., Boston, MA, USA. (Taoshu Biotechnology Co., Ltd., Shanghai, China) underwent detailed assessment via SPR spectroscopy to quantify real-time binding kinetics and equilibrium dissociation constants between the ligands and NP.

Subsequently, we predicted the pharmacological properties of compounds **1**–**16**. In this study, the ADMET descriptors algorithm and toxicity prediction (extensible) module of Discovery Studio v3.0 (Accelrys, San Diego, CA, USA) were used to predicate the ADMET properties. The ADMET prediction and toxicity predictions results are enlisted in Tables S2 and S3. We noticed that all compounds had acceptable solubility and absorption compared with nucleozin, according to the results of the ADME absorption level and toxicity probability (Table S2), which were in line with the results observed during the experiment. The drug likeness of compounds **1**–**16** was also evaluated using the filter by the Lipinski and Veber Rules module in Discovery Studio v3.0 (Accelrys, USA). The results of Lipinski’s rule calculation for compounds **1**–**16** indicated that the 16 compounds were in line with the Lipinski’s rule (Table S4).

### 2.2. Analysis of In Vitro Assays

**Figure 5:**
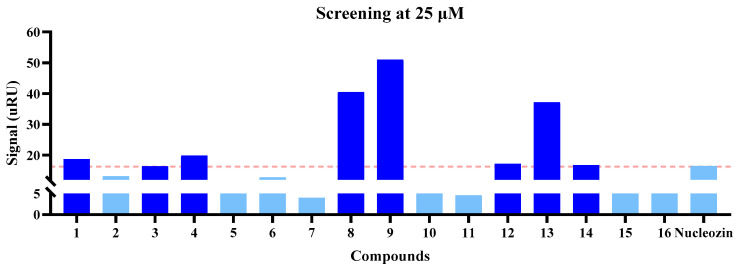
The binding affinities of selected compounds at 25 μM. Compounds are colored relative to the nucleozin signal (orange dashed line): dark blue for signals close to or above, and light blue for signals below the reference.

**Figure 6:**
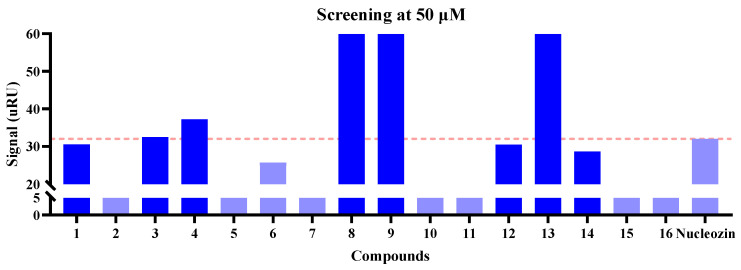
The binding affinities of selected compounds at 50 μM. Compounds are colored relative to the nucleozin signal (orange dashed line): dark blue for signals close to or above, and light blue for signals below the reference.

**Figure 7:**
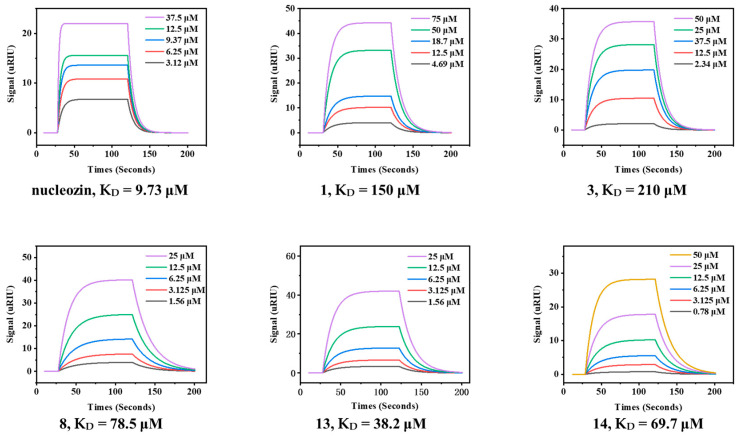
The binding affinities of the six potent NP inhibitors.

SPR analysis was performed to assess the molecular interactions between 16 lead compounds (purchased from TargetMol Chemicals Inc.) and their biological target, with detailed binding parameters documented in Figure 5 and Figure 6 and Table 2. We first screened the binding affinities between NP and the compounds at 25 and 50 μM. At 25 μM, compounds **1**, **3**, **4**, **8**, **9**, **12**, **13**, and **14** potentially exhibited a higher binding affinity for NP than nucleozin (Figure 5), a trend that was consistent with the observations at 50 μM (Figure 6). Notably, compounds **8**, **13**, and **14** demonstrated superior target engagement, showing equilibrium dissociation constants (*K*_D_) of 7.85 × 10^−5^ M, 3.82 × 10^−5^ M, and 6.97 × 10^−5^ M, respectively (Figure 7 and Table 2). Although the *K*_D_ values of these compounds were lower than nucleozin (*K*_D_ = 9.73 μM), these five candidates emerged as promising NP-targeting agents with novel scaffolds.

### 2.3. Molecular Dynamics Simulations

**Figure 8:**
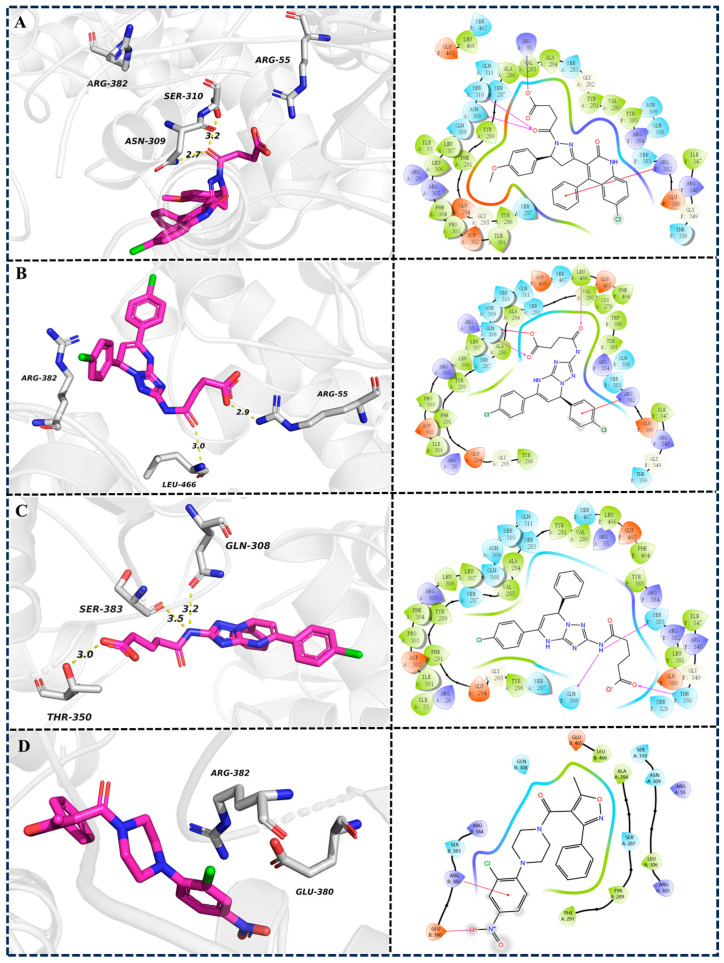
The docking poses of 5B7B-**8** (**A**), 5B7B-1**3** (**B**), 5B7B-**14** (**C**), and 5B7B–nucleozin (**D**). In the 3D diagram, the yellow dashed lines represent hydrogen bond interactions. In the 2D diagram, the purple arrows represent hydrogen bond interactions, and the red lines represent π–cation interaction.

Molecular docking analysis revealed distinct ligand–protein interaction patterns (Figure 8). Analysis of the binding modes revealed that compounds **8**, **13**, and **14** and nucleozin interact with 5B7B via a common binding interface, as revealed by the involvement of residues ALA284(A)-SER287(A)-TYR289(A)-PHE291(A), ARG305(A)-LEU306(A)-ASN309(A)-SER310(A), and GLU380(B)-ARG382(B)-SER383(B)-ARG384(B). Compound **8** formed hydrogen bonds with SER310(A) and ASN309(A) (Figure 8A), while compound **13** formed a hydrogen bond with ARG55(A) (Figure 8B). Compound **14** engaged in hydrogen bonding with GLN308(B), THR350(B), and SER383(B) (Figure 8C). Nucleozin formed a salt bridge with GLU380(B) (Figure 8D).

#### 2.3.1. Stability of Dynamics Trajectory from Root Mean Square Deviation (RMSD) Analysis

**Figure 9:**
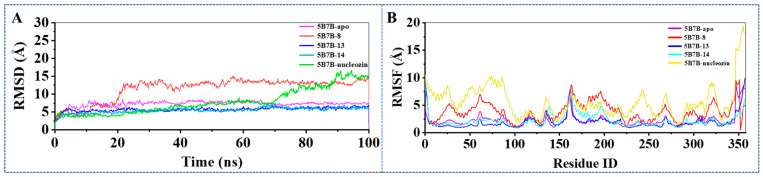
(**A**) RMSD of 5B7B-apo, 5B7B-**8**, 5B7B-**13**, 5B7B-**14**, and 5B7B–nucleozin during the 100 ns MD simulations. (**B**) RMSF of 5B7B-apo, 5B7B-**8**, 5B7B-**13**, 5B7B-**14**, and 5B7B–nucleozin during the 100 ns MD simulations.

In this investigation, Desmond software version 3.7 was employed to conduct 100 ns molecular dynamics simulations on three protein–ligand structures derived from docking studies, with the objectives of examining the interaction stability between biomolecules and investigating the functional roles of essential residues within the catalytic site. The dynamic behavior of these complexes was analyzed through RMSD trajectory measurements, where values of 5B7B-apo, 5B7B-**13**, and 5B7B-**14** were within 6 Å and maintained as the stability criterion throughout the simulation period, consistent with conventional standards for biomolecular complex stability assessment (Figure 9A). The backbone RMSD trajectories of 5B7B-**13** and 5B7B-**14** complexes converged following an initial 10 ns equilibration phase, with atomic positional variations remaining below 5 Å. 5B7B-**13** and 5B7B-**14** maintained average RMSD values of approximately 5 Å, demonstrating greater conformational stability than the 5B7B reference system throughout the entire 100 ns simulation trajectory. In contrast, the 5B7B-**8** and 5B7B–nucleozin complex exhibited persistent conformational instability throughout the entire 100 ns molecular dynamics simulation period. These observations collectively validate the attainment of energetically stable binding configurations during the dynamical sampling process.

#### 2.3.2. Structural Flexibility Evaluation from Root Mean Square Fluctuation (RMSF) Analysis

The structural integrity of protein–ligand complexes is critically governed by the dynamic behavior of constituent amino acid residues. This molecular mobility is quantitatively characterized through RMSF analysis, a computational metric that calculates the time-averaged positional displacement of individual residues relative to their equilibrium coordinates. Notably, RMSF profiling enables precise identification of localized flexibility hotspots within the protein architecture. Regions exhibiting elevated RMSF values correspond to residues with pronounced conformational plasticity, demonstrating substantial positional variance during trajectory sampling. Conversely, residues constrained to a low RMSF threshold maintain restricted mobility, reflecting their thermodynamic stabilization within the binding pocket microenvironment. As depicted in Figure 9B, the RMSF profiles of all four complexes during the 100 ns simulation displayed analogous dynamic patterns, compared with 5B7B-apo, 5B7B-**8**, and 5B7B–nucleozin systems, which demonstrated enhanced residue mobility relative to the 5B7B-**13** and 5B7B-**14** complexes. This differential flexibility implied that compounds **13** and **14** established more robust molecular interactions with the 5B7B system, effectively reducing conformational variability through enhanced structural stabilization.

#### 2.3.3. Intermolecular Interaction Analysis During the 100 ns MD Simulation

**Figure 10:**
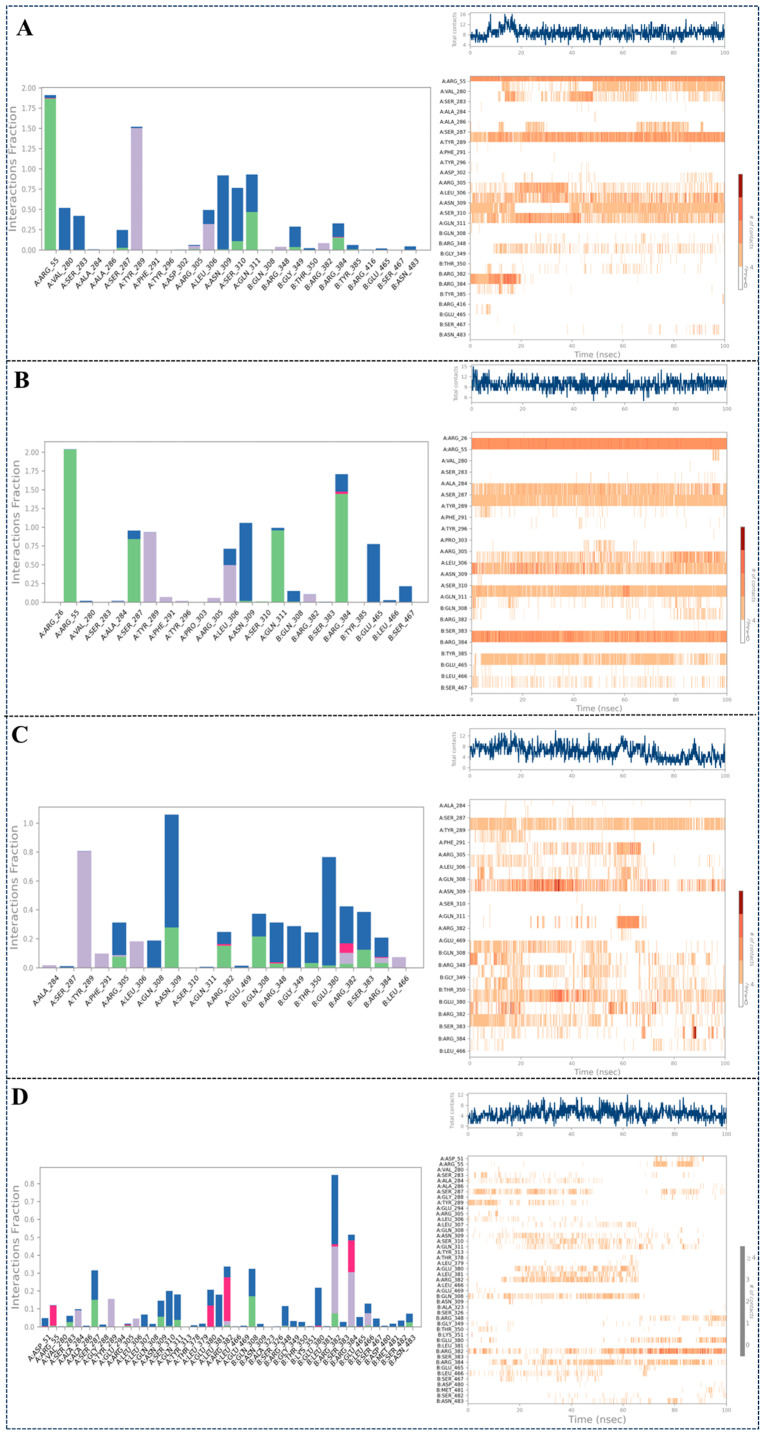
Protein–ligand contacts of protein–ligand during the simulation. The abscissa of the cumulated histogram indicates the vital residues that participate in the interactions, while the ordinate denotes interactions percentage. The interactions fraction is calculated depending on their duration percentage along the whole 100 ns MD simulations trajectory. The green histogram represents the hydrogen bonds; the purple histogram represents the hydrophobic interactions; the pink histogram represents the ionic bridges; the blue histogram represents the water bridges. (**A**) Protein-ligand contacts of 5B7B-**8** during the simulation; (**B**) Protein-ligand contacts of 5B7B-**13** during the simulation; (**C**) Protein-ligand contacts of 5B7B-**14** during the simulation; (**D**) Protein-ligand contacts of 5B7B-nucleozin during the simulation.

MD simulations provide enhanced resolution in studying protein–ligand binding conformations. During a 100 ns MD trajectory, four principal interaction categories were examined: hydrogen bonds, hydrophobic interactions, ionic bridges, and water bridges. Persistent interactions exceeding 30% temporal occupancy were mapped for visualization. Figure 10 presents a composite interaction profile alongside quantitative contact statistics between the macromolecule and ligand. The visualization employs a chromatic gradient where intensified orange hues correlate with elevated contact frequencies from ligand moieties, as quantified by the reference scale. As shown in Figure 10A, compound **8** exhibited key interactions with ARG55(A), VAL280(A), SER283(A), TYR289(A), LEU306(A), ASN309(A), SER310(A), GLN311(A), and ARG384(B) throughout the 100 ns molecular dynamics simulation. In Figure 10B, compound **13** displayed critical interactions with ARG55(A), SER287(A), TYR289(A), LEU306(A), ASN309(A), GLN311(A), ARG384(B), and GLU465(B) during the entire 100 ns simulation. Figure 10C illustrates that compound **14** formed essential interactions with residues TYR289(A), ARG305(A), LEU306(A), ASN309(A), GLN308(B), GLU380(B), and ARG384(B) over the 100 ns trajectory. In Figure 10D, compound nucleozin displayed critical interactions with SER287(A), TYR289(A), LEU306(A), ASN309(A), SER310(A), GLN311(A), GLU380(A), ARG382(A), GLN308(B) GLU380(B), ARG382(B), and AGR384(B) during the entire 100 ns simulation. Notably, TYR289(A), LEU306(A), ASN309(A), and ARG384(B) consistently participated in binding across all simulations, highlighting their critical role in stabilizing protein–ligand interactions. In contrast, other amino acids involved in interactions monitoring exhibit significant fluctuations over the 100 ns period, suggesting that they might be non-essential.

#### 2.3.4. Analysis of Ligand Properties During the 100 ns MD Simulation

**Figure 11:**
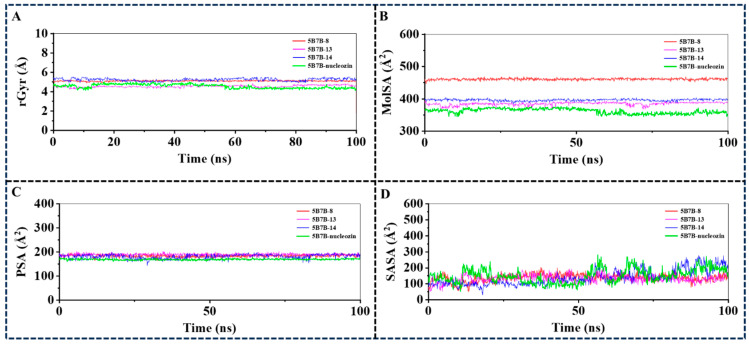
Ligand properties during the 100 ns MD simulations. (**A**) Radius of Gyration of 5B7B-**8**, 5B7B-**13**, 5B7B-**14**, and 5B7B-nucleozin during the 100 ns MD simulations; (**B**) Molecular Surface Area of 5B7B-**8**, 5B7B-**13**, 5B7B-**14**, and 5B7B-nucleozin during the 100 ns MD simulations; (**C**) Polar Surface Area of 5B7B-**8**, 5B7B-**13**, 5B7B-**14**, and 5B7B-nucleozin during the 100 ns MD simulations; (**D**) Solvent Accessible Surface Area of 5B7B-**8**, 5B7B-**13**, 5B7B-**14**, and 5B7B-nucleozin during the 100 ns MD simulations.

Radius of Gyration (rGyr), Molecular Surface Area (MolSA), Solvent Accessible Surface Area (SASA), and Polar Surface Area (PSA) serve as critical metrics in conformational analysis plots, offering complementary analytical perspectives on protein–ligand binding mechanisms. All ligands exhibited rGyr values approximating 5 Å (Figure 11A), suggesting the presence of an expansive binding pocket, which may warrant the structural incorporation of auxiliary functional groups in pharmacophore optimization. The MolSA profiles mirrored the van der Waals surface characteristics, with compounds **13** and **14** displaying minimal divergence in these measurements (Figure 11B). The PSA and SASA trends aligned with intermolecular forces identified in prior docking analyses and dynamic simulations (Figure 11C,D). Collectively, these ligand–protein interfacial properties may serve as valuable structural determinants for rational design and refinement of next-generation NP inhibitors.

### 2.4. Alanine Scanning Mutagenesis

**Figure 12:**
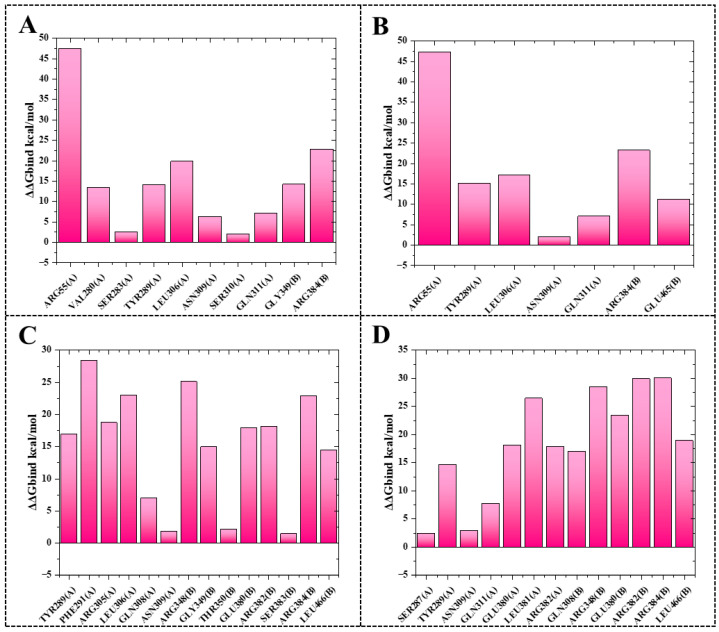
Alanine scanning mutagenesis analysis of the crucial amino acids in protein–ligand complexes 5B7B-**8** (**A**), 5B7B-**13** (**B**), 5B7B-**14** (**C**), and 5B7B–nucleozin (**D**).

To further confirm the proposed interaction mechanism, systematic alanine substitution experiments were conducted on key residues in three protein–ligand complexes (5B7B-**8** (Figure 12A), 5B7B-**13** (Figure 12B), 5B7B-**14** (Figure 12C), and 5B7B–nucleozin (Figure 12D)). The replacement of TYR289(A), ASN309(A), and ARG384(B) with alanine consistently destabilized the molecular systems, as evidenced by elevated binding free energy changes (ΔΔGbind). This demonstrates the critical functional importance of these residues’ side chain groups in maintaining stable ligand–receptor interactions.

### 2.5. Quantum Mechanics/Molecular Mechanics (QM/MM) Analysis

**Figure 13:**
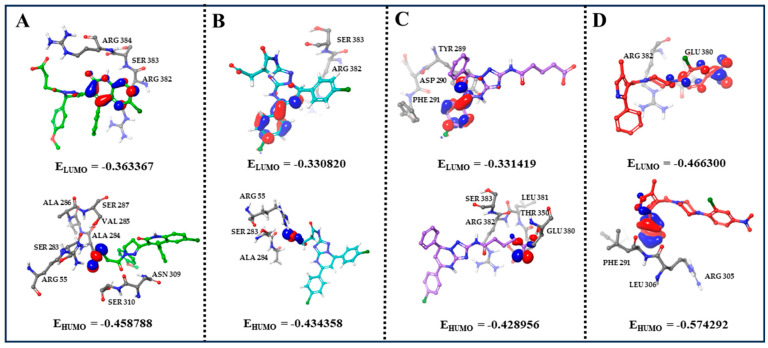
The HOMO and LUMO analysis by QM/MM analysis. (**A**) The HOMO and LUMO analysis of **8**. (**B**) The HOMO and LUMO analysis of **13**. (**C**) The HOMO and LUMO analysis of **14**. (**D**) The HOMO and LUMO analysis of nucleozin.

We computed the highest occupied molecular orbital (HOMO) and the lowest unoccupied molecular orbital (LUMO) to characterize the pi-pi stacking and electrostatic interactions within the system. Typically, the π orbitals of aromatic rings correspond to the HOMO and LUMO, which mediate pi-pi stacking interactions. The interaction between aromatic rings is primarily driven by their π-electron clouds. The HOMO, having higher electron density, generally displays a partial negative charge. This facilitates interactions with positively charged amino acid side chains, such as lysine and arginine. Conversely, the LUMO possesses strong electron-accepting capability and can exhibit a partial positive charge, enabling interactions with negatively charged side chains like glutamate and aspartate. Both HOMO and LUMO orbitals play crucial roles in mediating drug–amino acid interactions. For compound **8**, the LUMO resides within the quinolyl group surrounded by ARG382, SER383, and ARG384, while the HOMO is distributed across the carboxyl group located in the amino acid residues ARG55, SER283, ALA284, VAL285, ALA286, SER287, ASN309, and AER310 (Figure 13A). For compound **13**, the LUMO is localized on the phenyl group, and the main interacting amino acid residues are ARG382 and SER383. The HOMO is on the carboxyl group, which is surrounded by ARG55, SER283, and ALA284 (Figure 13B). For compound **14**, the LUMO is found on the phenyl group and located in TYR289, ASP290, and PHE291. The HOMO on the carboxyl group is surrounded by THR350, GLU380, LEU381, ARG382, and SER383 (Figure 13C). For nucleozin, the LUMO resides within the nitro group and generates interactions with ARG382 and GLU380, while the HOMO is distributed across the phenyl group and surrounded by PHE291, ARG305, and LEU306 (Figure 13D). These findings are consistent with the pi-pi stacking and electrostatic interactions identified in our molecular docking and molecular dynamics simulation data.

### 2.6. Dynamic Cross-Correlation Matrix (DCCM) Analysis and Principal Component Analysis (PCA)

**Figure 14:**
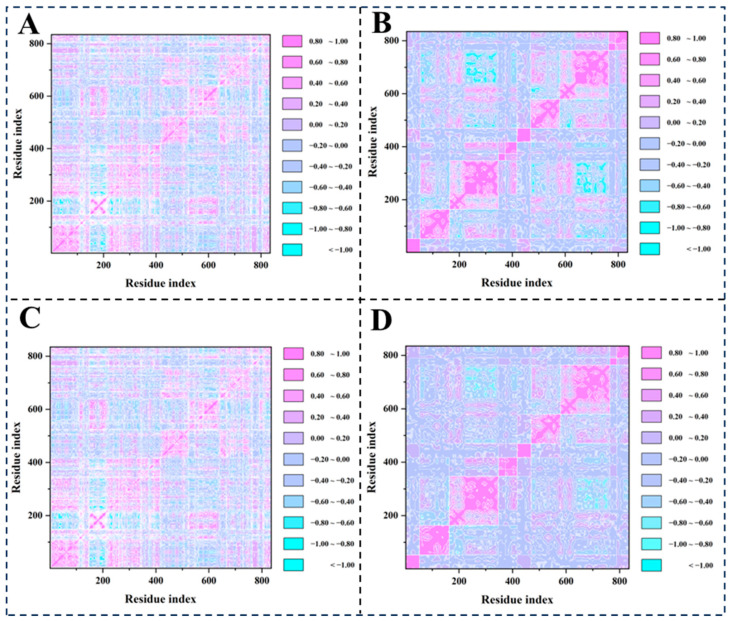
DCCM analysis of 5B7B-**8** (**A**), 5B7B-**13** (**B**), 5B7B-**14** (**C**), and 5B7B–nucleozin (**D**). Strong correlated motions between the residues in 5B7B are represented as high positive regions (blue), while strong anti-correlated motions are depicted as negative regions (purple). The deeper the color, the larger the Cij values.

DCCM analysis effectively identifies functionally coupled regions within the protein structure (Figure 14A–D). Notably, residues in the ligand binding pocket display high correlation values, reflecting their coordinated motion required for ligand binding. The analysis uncovers distinct dynamic coupling patterns between compounds (**8**, **13**, **14**, and nucleozin) and the NP binding pocket. All three compounds exhibit heterogeneous correlation landscapes featuring prominent cyan (positive) and pink (negative) regions, signifying significant allosteric modulation of residue–residue couplings.

PCA plots (Figure S1) depict the conformational landscape of the NP (PDB ID: 5B7B) bound to ligands **8**, **13**, **14**, and nucleozin (states 5B7B-**8**, 5B7B-**13**, 5B7B-**14**, and 5B7B–nucleozin). Projections onto the first two principal components (PC1: x-axis, PC2: y-axis) illustrate the system’s dynamics. The variance explained by PC1 and PC2 for each state is 23.25% and 8.29% for 5B7B-**8**, 17.58% and 10.42% for 5B7B-**13**, 39.63% and 9.77% for 5B7B-**14**, and 17.65% and 10.53% for 5B7B–nucleozin. In all states, trajectory points are color-graded from blue (0 ns) to red (100 ns), revealing a distinct temporal evolution. The substantial combined variance explained by PC1 and PC2 highlights significant structural heterogeneity within each protein–ligand complex. Notably, the variance distribution in 5B7B-**14** diverges somewhat, potentially reflecting differences in experimental parameters or ligand binding kinetics. Clustering of points along the eigenvectors demonstrates time-dependent groupings. Segregation between early-stage (pink) and late-stage (cyan) conformations into distinct regions is evident. This separation indicates that the binding of ligands **8**, **13**, and **14** triggers detectable conformational changes in NP subunits, with the majority of this structural variance captured by the first two principal components in each analysis.

## 3. Materials and Methods

### 3.1. Virtual Screening

The virtual screening campaign leveraged over 10 million small molecules, with the majority procured from Chemdiv and Chembridge’s proprietary chemical repositories. Compound preprocessing, including tautomeric state assignment and structural refinement, was performed through Maestro’s LigPre module, using the OPLS3e force field for molecular mechanics optimization. The process of virtual screening and its subsequent refinement was conducted utilizing the Glide module within the Schrödinger Maestro software suite (version 2023-1). The crystal structure of NP (PDB ID: 5B7B) was subjected to structural refinement and minimization through the protein preparation wizard module. All compounds were prepared in accordance with the default parameters established by the LigPre module. The refined NP structure was then imported into the Glide module for the screening process. Docking sites were identified based on the bound ligand nucleozin, which served as the centroid for 18 Å box. To validate the docking methodology, the ligand was redocked with RMSD lower than 1.5 Å. The dataset underwent screening via HTSV and SP docking techniques. Following this, the XP docking template was employed to evaluate pro-ligands that exhibited higher scores, as determined by the SP method. The selection of the XP method was aimed at enhancing the correlation between docking poses and scoring, thereby improving the overall accuracy of the results.

Compound prioritization integrated multi-parametric evaluation criteria beyond the GlideScore rankings, including pharmacophoric features analysis and binding free energy decomposition. Critical biomolecular recognition events—spanning hydrogen bond networks, π-π stacking, and hydrophobic complementarity—were quantitatively assessed using Schrödinger’s Interaction Fingerprint module.

### 3.2. Prime/MM–GBSA Simulation

The ligand binding free energies (ΔG_bind_) were computed using the MM/GBSA implicit solvent model, as defined by the thermodynamic cycle [30]:

ΔG_bind_ = ΔE_MM_ + ΔG_solv_ + ΔG_SA_ (1)

In this formulation, ΔE_MM_ quantifies the potential energy difference between the geometrically optimized ligand–receptor complex and the sum of the gas-phase potential energies of the isolated ligand and protein components. ΔG_solv_ represents the net solvation free energy difference between the ligand–protein complex and the sum of solvation energies for the dissociated ligand and protein components, as computed through the GBSA implicit solvent model. The term ΔG_SA_ quantifies the discrepancy in surface energy between the assembled protein–ligand complex and the combined surface energy values of the isolated protein and ligand components.

### 3.3. Lipinski’s Rule and ADMET Prediction

ADMET characteristics constitute essential selection criteria within current drug development pipelines. We systematically determined fundamental pharmacokinetic parameters by employing Discovery Studio v3.0 (Accelrys, USA), including BBB permeability, gastrointestinal absorption efficiency, aqueous solubility indices, hepatotoxic potential, and plasma protein binding affinity. Compound safety profiles were subsequently predicted through toxicological assessment via the TOPKAT module. Drug-like properties were further validated using an integrated computational approach encompassing Lipinski’s Rule of Five and Veber’s bioavailability parameters.

### 3.4. Materials

The 16 hit compounds were purchased from TargetMol Chemicals Inc. (Taoshu Biotechnology Co., Ltd. Shanghai, China).

### 3.5. Expression and Purification of NP

The NP gene was subjected to double digestion using EcoRI and HindIII restriction enzymes. Recombinant NP protein, featuring an N-terminal hexahistidine tag, was overexpressed in *Escherichia coli* BL21(DE3) pLysS. Bacterial cultures were grown in LB medium containing kanamycin and chloramphenicol. Protein expression was induced at an OD600 of 0.6–0.8 by adding IPTG to a final concentration of 0.4 mM. Following induction, cultures were incubated at 21 °C with shaking at 210 rpm for 12–16 h before cells were collected via centrifugation. The cell pellet was resuspended and lysed by sonication in chilled NP lysis buffer [20 mM sodium phosphate pH 7, 1.5 M NaCl, 1 mM phenylmethylsulfonyl fluoride (PMSF)]. The lysate was clarified by centrifugation at 21,000× *g* for 1 h at 4 °C. The supernatant was applied to a nickel affinity column. After washing with NP lysis buffer, bovine pancreatic RNase A (Sigma, St. Louis, MO, USA) was introduced at 1 U/mL to eliminate residual *Escherichia coli* RNA. The nickel affinity resin was incubated for 1 h at room temperature with agitation, followed by extensive washing with NP lysis buffer containing 50 mM imidazole. Bound proteins were eluted using NP elution buffer (20 mM sodium phosphate pH 7, 150 mM NaCl, 500 mM imidazole). Further purification was carried out using heparin affinity chromatography, with elution performed with 20 mM sodium phosphate pH 7 containing 1.5 M NaCl. For proteins intended for crystallization screening, an additional size-exclusion chromatography step was implemented using a Superdex 200 column (GE Healthcare, Chicago, IL, USA) equilibrated with 20 mM 3-(*N*-morpholino)propanesulfonic acid (MOPS) pH 7 and 150 mM NaCl.

### 3.6. SPR Experiment

SPR-based affinity assessments could be performed through the Reichert 2SPR (Reichert-Ametek, Buffalo, NY, USA). First, the sensor chip coated with carboxymethyl dextran hydrogel was secured, and its surface was activated using a mixture of EDC and NHS. Following immobilization of NP in 10 mM sodium acetate buffer (pH 4.5), channel 1 exhibited an RU value of 7000 specific to the protein. The protein was blocked using 1M ethanolamine at pH 8. The *K*_D_ values were determined using a compound dilution series (0.78 μM to 200 μM) applied across the sensor chip. The affinity fit was calculated with the Trace Drawer software (version 1.8) through nonlinear regression analysis.

### 3.7. Molecular Dynamics Simulation

The molecular dynamics simulations of protein–ligand systems were executed with Desmond (version 3.7) software [31,32,33]. These simulations applied the OPLS3e force field, incorporated the TIP3 solvent model for hydration, and introduced counterions to achieve charge balance in the system. The full molecular assembly underwent energy refinement employing the all-atom OPLS3e force field. Structural rigidity was enforced through the SHAKE protocol, maintaining fixed geometries for aqueous species while preserving rigid bond parameters (lengths/angles) in non-hydrogen atoms. Periodic boundary constraints were implemented throughout the simulations, effectively replicating bulk-phase conditions by eliminating finite system artifacts. Electrostatic interactions at extended ranges were processed through the Particle Mesh Ewald (PME) summation technique. System equilibration followed an isothermal–isobaric (NPT) protocol with thermodynamic setpoints fixed at 300 K and 1.0 bar, utilizing the Berendsen thermostat barostat apparatus for simultaneous thermal and mechanical regulation. Finalized system configuration involved executing a production-level simulation spanning 200 nanoseconds with 1.2-femtosecond temporal resolution. Conformational sampling was systematically captured at 50-picosecond intervals, yielding 4 × 10^4^ trajectory frames for subsequent analysis. Backbone coordinate stability was quantitatively evaluated through RMSD metrics, complemented by molecular interaction mapping to characterize ligand binding interface dynamics.

### 3.8. Alanine Scanning Mutagenesis

Amino acid substitution mutagenesis (ASM) serves as a fundamental experimental strategy in molecular interrogation frameworks, enabling systematic probing of structural–electronic contributions from discrete residue positions during biomolecular recognition events at intermolecular interfaces [30,34,35]. This approach functions as an essential verification scaffold for computational predictions derived from energetic partitioning schemes. The thermodynamic perturbation magnitude (ΔΔG_bind_) is quantitatively parameterized through the implementation of this mathematical formalism:

ΔΔG_bind_ = ΔG_bind, mutant_ − ΔG_bind, wild type_ (2)

The ΔΔG_bind_ parameter, induced by alanine scanning mutagenesis, is operationally defined as the binding free energy differential between the wild type (ΔG_bind, wild type_) and the mutant (ΔG_bind, mutant_). Elevated ΔΔG_bind_ magnitudes directly correlate with the mutation’s capacity to induce greater energetic destabilization, providing thermodynamic validation of the specific residue’s role as an energetic determinant in molecular recognition processes [30].

### 3.9. QM/MM Calculations

QM/MM computations were executed within the Schrödinger computational platform (version 2023-1), leveraging the QSite module’s quantum embedding architecture. Target molecular motifs were explicitly modeled at the DFT level (quantum subsystem), while peripheral structural elements—including the protein scaffold, solvation shell, and counterion network—were governed by the OPLS4 empirical potential [36]. The QM/MM boundary transitions were implemented via hydrogen-capping methodology to maintain electronic continuity [37]. The QM subsystem was resolved using dispersion-corrected Density Functional Theory (DFT) with the B3LYP-D3 functional [38] and 6-31G** basis functions. Atoms with significant mass located in the MM zone farther than 15 Å from the QM boundary were subjected to harmonic restraints (spring constant: 0.5 kcal/mol·Å22). For mapping the Potential Energy Surface (PES), critical reaction pathways were determined, followed by unconstrained PES scans to estimate the positions of tentative transition states. Transition state geometries were optimized through QST3 computational methodology, with subsequent vibrational frequency calculations to validate the presence of a sole imaginary frequency. Essential reaction pathways underwent thermodynamic characterization via umbrella sampling simulations along predefined coordinates (50 sampling intervals, 100 ps per trajectory segment). The potential of mean force was derived through WHAM statistical processing. Electronic charge allocation, covalent bonding characteristics, and frontier orbital couplings within the quantum domain were investigated using the natural bond orbital (NBO) computational framework.

### 3.10. DCCM Analysis and PCA

A DCCM was generated to quantify residue-level conformational coupling patterns derived from the MD trajectories. Pairwise dynamic correlations among Cα atomic positions were calculated via the Bio3d computational toolkit, leveraging trajectory data to map coordinated movements [39]. To reduce data variability and account for structural differences in side chain conformations, our study focused exclusively on the Cα atomic coordinates within protein residues. Inter-residue correlations were analyzed through covariance calculations, with matrix components Cij representing interaction intensities determined by the following formula:

Cij = Δri × Δrj/(Δri^2^ × Δrj^2^)^1/2^ (3)

Here, Δri and Δrj correspond to the instantaneous displacements of atomic positions from their equilibrium states along the simulation timeline, with angle brackets designating the time-evolved ensemble averaging across molecular dynamics trajectories. The normalized covariance coefficient Cij ranges between −1.0 (perfectly out-of-phase dynamics) and +1.0 (fully in-phase motions), establishing a metric for residue-specific dynamical coupling within the protein structure.

Furthermore, PCA was carried out with the Bio3d library in R (version 3.1.0) to analyze conformational dynamics derived from molecular dynamics simulation trajectories.

## 4. Conclusions

This study successfully identified three novel NP inhibitors (**8**, **13**, and **14**) through an integrated computational and experimental approach. Virtual screening of over 10 million compounds against the NP binding pocket (PDB ID: 5B7B) prioritized candidates with high binding affinity, as validated by SPR assays. The *K*_D_ values for these compounds were determined to be 7.85 × 10^−5^ M, 3.82 × 10^−5^ M, and 6.97 × 10^−5^ M, respectively, demonstrating sub-micromolar affinity, which indicates that the three candidates possess novel structural scaffolds and promising NP-targeting agents. These findings highlight the efficacy of structure-based drug discovery in targeting conserved viral proteins for antiviral development.

Molecular dynamics simulations and QM/MM analysis provided atomic-level insights into the binding mechanisms. Four key residues (TYR289, LEU306, ASN309, and ARG384) were identified as critical for stable interactions, forming hydrogen bonds, hydrophobic contacts, and water bridges. Alanine scanning mutagenesis confirmed the functional importance of these residues, with significant increases in binding free energy upon mutation. The electronic properties, such as HOMO-LUMO distributions, further elucidated the role of π-π stacking and electrostatic forces in facilitating ligand–receptor binding. This mechanistic understanding offers a foundation for rational optimization of NP inhibitors.

The robustness of the applied workflow, combining virtual screening, molecular dynamics simulations, and experimental validation, underscores its utility in accelerating drug discovery. This integrated pipeline not only efficiently narrowed down potential hits from a vast chemical library but also provided a comprehensive assessment of binding stability and specificity through biophysical and computational methods. The approach minimizes resource-intensive trial-and-error, making it a valuable strategy for identifying lead compounds against emerging viral targets. These results reinforce NP as a promising druggable target due to its high conservation across influenza strains and lack of human homologs, reducing the risk of resistance development. The identified inhibitors exhibit potential for broad-spectrum activity against influenza A and B viruses, paving the way for novel therapeutics that disrupt viral replication. Future work should focus on optimizing these compounds through residue-directed modifications and advancing them to in vivo efficacy studies to combat seasonal and pandemic influenza threats.

## Figures and Tables

**Table 1:** Glide score, MMGBSA, and binding free energies of selected 16 compounds.

No.	Glide Score	MMGBSA ΔG_Bind_ (kcal/mol)	MMGBSA ΔG_Bind vdW_ (kcal/mol)	MMGBSA ΔG_Bind Solv GB_ (kcal/mol)
nucleozin	−4.835	−41.83	−47.54	9.19
**1**	−7.832	−38.95	−50.49	109.15
**2**	−6.515	−37.77	−40.26	105.12
**3**	−6.341	−36.90	−55.11	409.67
**4**	−6.572	−52.60	−61.46	99.66
**5**	−6.912	−35.32	−58.04	102.68
**6**	−6.246	−57.63	−62.54	39.88
**7**	−6.436	−44.76	−53.07	239.10
**8**	−10.940	−46.79	−59.25	108.27
**9**	−6.653	−39.94	−57.16	211.07
**10**	−6.648	−55.10	−62.14	95.06
**11**	−6.473	−51.54	−50.27	100.60
**12**	−6.060	−44.21	−52.59	210.74
**13**	−7.161	−52.84	−54.04	105.39
**14**	−5.560	−50.16	−53.87	119.75
**15**	−6.654	−68.02	−67.37	36.61
**16**	−5.492	−59.16	−45.97	25.81

**Table 2:** The binding affinities of selected compounds.

Compd.	*K*_D_ (M)	Compd.	*K*_D_ (M)	Compd.	*K*_D_ (M)
nucleozin	9.73 × 10^−6^	**3**	2.10 × 10^−4^	**13**	3.82 × 10^−5^
**1**	1.50 × 10^−4^	**8**	7.85 × 10^−5^	**14**	6.97 × 10^−5^

## Data Availability

Data are contained within the article.

## Supplementary Materials

The following supporting information can be downloaded at: https://www.mdpi.com/article/10.3390/molecules30193960/s1. The structures, estimated activity, specific pharmacophore mapping, and docking poses of the hit compounds were supported in Supplemental Tables S1–S4 and Figure S1. Table S1. The selected 16 compounds; Table S2. The ADMET prediction results for compounds **1**–**16** and nucleozine.; Table S3. Toxicity Predictions of compounds **1**–**16** and nucleozine; Table S4. The results of Lipinski’s rule calculation for compounds **1**–**16** and nucleozine; Figure S1. PCA Analysis for 5B7B-**8** (A), 5B7B-**13** (B), 5B7B-**14** (C) and 5B7B-nucleozin (D).
