# Supplementary material for: Computational Discovery of Potent Nucleoprotein Inhibitors for Influenza A Virus: Validation Through QM/MM Analysis and Experimental Binding Assays
Source: Molecules. 2025 Oct 2;30(19):3960. doi: 10.3390/molecules30193960 (PMC12525657; doi:10.3390/molecules30193960)
Supplement: Supplementary file 1 [file molecules-30-03960-s001.zip › molecules-3834096-supplementary.pdf]

Computational Discovery of Potent Nucleoprotein Inhibitors for Influenza A Virus: Validation through QM/MM Analysis and Experimental Binding Assays

Zixiao Liu [1,2,†], Jialin Guo [2,†], Chao Zhang [2], Yongzhao Ding [1,2], Shiyang Sun [2], Binrong Yao [2], Cheng Xing [2], Xiaoping Liu [1,*], Chun Hu [1] and Junhai Xiao [2,*]

Table S1. The selected 16 compounds.

No.	Structure	MW	Formula	Glide Score	MMGBSA ΔG_{Bind}
1	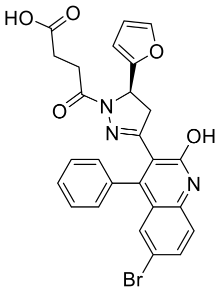	534.366	$C_{26}H_{20}BrN_3O_5$	-7.832	-38.95
2	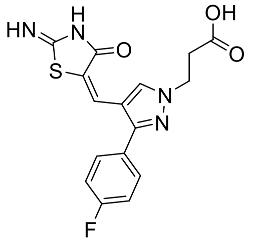	360.363	$C_{16}H_{13}FN_4O_3S$	-6.515	-37.77
3	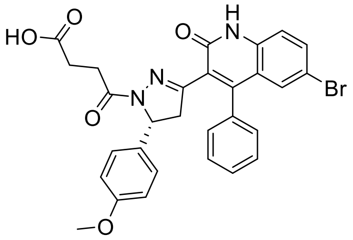	574.431	$C_{29}H_{24}BrN_3O_5$	-6.341	-36.90
4	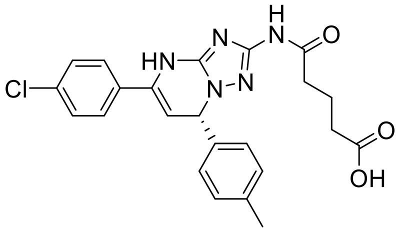	451.911	$C_{23}H_{22}ClN_5O_3$	-6.572	-52.60
5	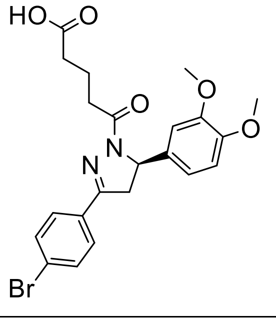	479.339	$C_{22}H_{23}BrN_2O_5$	-6.912	-35.32
6	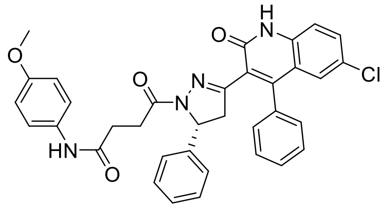	605.091	$C_{35}H_{29}ClN_4O_4$	-6.246	-57.63

7	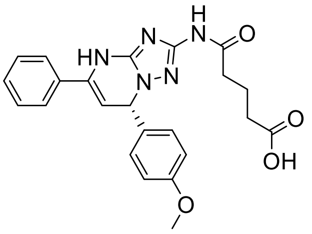	433.468	$C_{23}H_{23}N_5O_4$	-6.436	-44.76
8	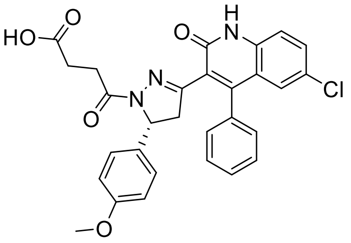	529.977	$C_{29}H_{24}ClN_3O_5$	-10.940	-46.79
9	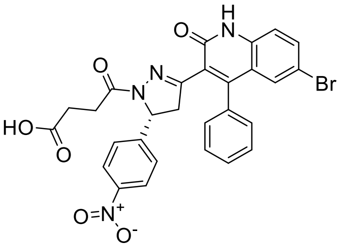	589.402	$C_{28}H_{21}BrN_4O_6$	-6.653	-39.94
10	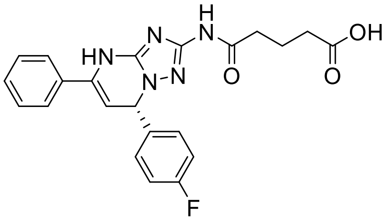	421.432	$C_{22}H_{20}FN_5O_3$	-6.648	-55.10
11	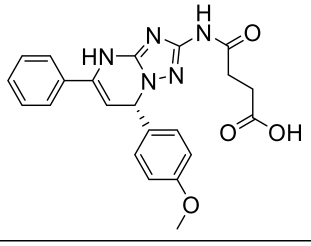	419.441	$C_{22}H_{21}N_5O_4$	-6.473	-51.54
12	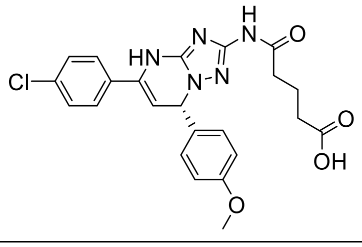	467.910	$C_{23}H_{22}ClN_5O_4$	-6.060	-44.21
13	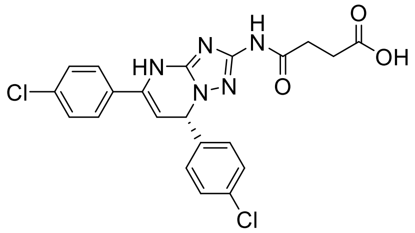	458.299	$C_{21}H_{17}Cl_2N_5O_3$	-7.161	-52.84

14	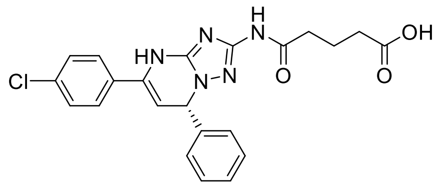	437.884	$C_{22}H_{20}ClN_5O_3$	-5.560	-50.16
15	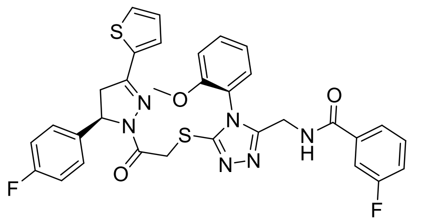	644.716	$C_{32}H_{26}F_2N_6O_3S_2$	-6.654	-68.02
16	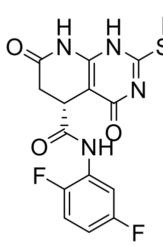	380.370	$C_{16}H_{14}F_2N_4O_3S$	-5.492	-59.16

Table S2. The ADMET prediction results for compounds **1-16** and nucleozine.

No.	ADME Solubility Level	ADME BBB Level	ADME Absorption Level	PPB Prediction	CYP2D6 Prediction
**1**	2	4	2	true	false
**2**	2	4	0	true	false
**3**	2	4	1	true	false
**4**	2	4	1	true	false
**5**	2	2	0	true	false
**6**	2	4	2	true	false
**7**	2	4	1	true	false
**8**	2	4	1	true	false
**9**	2	4	2	true	false
**10**	2	4	0	true	false
**11**	2	4	0	true	false
**12**	2	4	1	true	false
**13**	2	4	1	true	false
**14**	2	4	1	true	false
**15**	2	4	2	true	false
**16**	2	3	0	false	false
**Nucleozine**	3	3	0	true	false

ADME_Solubility_Level: 0 (Extremely low); 1 (No, very low, but possible); 2 (Yes, low); 3 (Yes, good); 4 (Yes, optimal); 5(No, too soluble); 6 (Warning: molecules with one or more unknown AlogP98 types).

ADME_BBB_Level: 0 (Very high penetrant); 1 (High); 2 (Medium); 3 (Low); 4 (Undefined).

ADME_Absorption_Level: 0 (Good absorption); 1 (Moderate absorption); 2 (Low absorption); 3 (Very low absorption).

EXT_PPB_Prediction: plasma protein binding ability, false: ≥ 90 %; true: ≤ 90 %.

EXT_CYP2D6_Prediction: false: non-inhibitor; true: inhibitor.

Table S3. Toxicity Predictions of compounds **1-16** and nucleozine.

No.	NTP carcinogenicity male Rat	NTP carcinogenicity female Rat	NTP carcinogenicity Call (Male mouse)	NTP carcinogenicity Call (Female mouse)	Ames mutagenicity	Rat oral LD50 (in g/kg)	Skin irritation
**1**	NC	NC	NC	NC	NM	0.504258	None
**2**	NC	NC	NC	NC	NM	0.684301	None
**3**	NC	NC	NC	NC	NM	3.37852	None
**4**	NC	NC	NC	NC	NM	4.5162	None
**5**	NC	C	NC	NC	NM	1.95438	None
**6**	NC	NC	C	NC	NM	5.61508	None
**7**	NC	NC	NC	NC	NM	1.53735	None
**8**	NC	NC	NC	NC	NM	1.70357	None
**9**	NC	NC	NC	NC	NM	25.8085	None
**10**	NC	NC	NC	NC	NM	0.863429	None
**11**	NC	NC	NC	NC	NM	1.34995	None
**12**	NC	NC	NC	NC	NM	1.08302	None
**13**	NC	NC	NC	NC	NM	1.97433	None
**14**	NC	NC	NC	NC	NM	1.67356	None
**15**	NC	NC	NC	NC	NM	0.37959	Mild
**16**	NC	NC	C	NC	NM	2.54227	None
**Nucleozine**	NC	NC	NC	NC	NM	8.22562	None

C: Carcinogen; NC: Non-Carcinogen; NM: Non-Mutagen.

Table S4. The results of Lipinski's rule calculation for compounds **1-16** and nucleozine.

No.	Alop	MW	No. HBA	No. HBD	No. rotatable bonds
**1**	4.635	534.36	6	2	6
**2**	2.528	360.36	6	3	5
**3**	4.18	574.42	6	2	7
**4**	4.354	451.91	6	3	7
**5**	3.769	475.33	6	1	8
**6**	5.251	605.08	5	2	8
**7**	3.187	433.46	7	3	8
**8**	4.096	529.97	6	2	7
**9**	2.948	590.40	7	3	7
**10**	3.409	421.42	6	3	7
**11**	2.73	419.43	7	3	7
**12**	3.851	467.90	7	3	8
**13**	4.076	458.30	6	3	6
**14**	3.867	437.88	6	3	7
**15**	5.83	644.71	7	1	10
**16**	1.641	380.37	6	3	4
**Nucleozin**	2.835	427.86	5	1	4

MW: molecular weight; HBA: H-bond acceptors; HBD: H-bond donors.

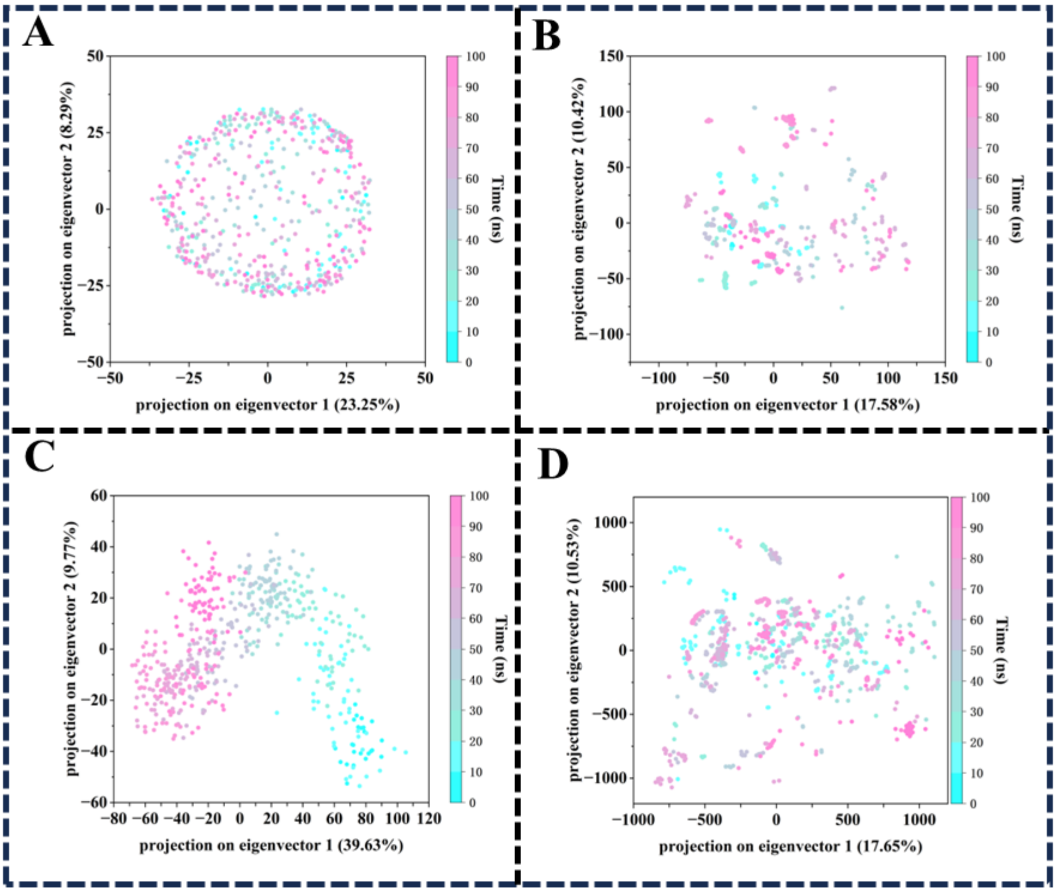

Figure S1. PCA Analysis for 5B7B-**8** (A), 5B7B-**13** (B), 5B7B-**14** (C) and 5B7B-nucleozin (D).
